# Bacterial foraging-optimized PID control of a two-wheeled machine with a two-directional handling mechanism

**DOI:** 10.1186/s40638-017-0057-3

**Published:** 2017-03-23

**Authors:** K. M. Goher, S. O. Fadlallah

**Affiliations:** 1 Department of Informatics and Enabling Technologies, Lincoln University, Lincoln, New Zealand; 2 Mechanical Engineering Department, Auckland University of Technology, Auckland, New Zealand

**Keywords:** Two-wheeled machine, Inverted pendulum, Two-directional handling, Lagrangian modeling, PID, BFO

## Abstract

This paper presents the performance of utilizing a bacterial foraging optimization algorithm on a PID control scheme for controlling a five DOF two-wheeled robotic machine with two-directional handling mechanism. The system under investigation provides solutions for industrial robotic applications that require a limited-space working environment. The system nonlinear mathematical model, derived using Lagrangian modeling approach, is simulated in MATLAB/Simulink^®^ environment. Bacterial foraging-optimized PID control with decoupled nature is designed and implemented. Various working scenarios with multiple initial conditions are used to test the robustness and the system performance. Simulation results revealed the effectiveness of the bacterial foraging-optimized PID control method in improving the system performance compared to the PID control scheme.

## Background

The interest in two-wheeled machines (TWMs) is incomparably increasing, and various linear and nonlinear methods of identification are employed for developing an accurate model of the inverted pendulum and establishing a proper control strategy for the system. Lee et al. [1] concentrated on designing a one-wheel inverted pendulum system that employs air power for balancing. The pitch angle was controlled by a DC motor, while the roll angle was regulated by air pressure sent out from ducted fans controlled by linear control methods. Chinnadurai and Ranganathan [2] focused on applying the principle of IP by proposing a two-wheel self-supporting robot controlled by an internet-on-a chip (IOC) controller. The main feature associated with this system is the capability to control the robot worldwide using the IOC, not to mention the IR, attitude, and tilt sensors installed on the robot. A novel configuration of wheeled robotic machines (WRM) which is based on the principle of two-wheeled inverted pendulum (IP) with an extended intermediate body (IB) was developed by Goher and Tokhi [3]. For providing multiple lifting levels for a carried payload, the developed machine is equipped with a linear actuator. The previously mentioned WRM was later improved by Almeshal et al. [4]. Increasing the machine’s flexibility and workspace led to a novel five DOFs two-wheeled IP with an extended rod.

### Implementation of optimization techniques on IP systems

Determining the optimal control strategy for IP systems has been and still a major concern for significant amount of studies. Diverse modeling techniques and control approaches have been applied for investigating and controlling this highly nonlinear system [5, 8]. Due to their outstanding successful applications in various areas of interest, nature-inspired and bio-inspired optimization algorithms are significantly gaining attention in nowadays research aspects. Within the past decade, there has been a tremendous amount of research studies focusing on developing optimization algorithms. Some of these algorithms include particle swarm optimization (PSO) algorithm [9], spiral dynamics algorithm (SDA) [10], genetic algorithm (GA) [11], and bacterial foraging optimization (BFO) [12].

## Bacterial foraging optimization (BFO) algorithm

The survival of species in any natural evolutionary process depends upon their fitness criteria, which relies upon their food searching and motile behavior. The law of evolution supports those species who have better food searching ability and either eliminates or reshapes those with poor search ability. The genes of those species who are stronger get propagated in the evolution chain since they possess ability to reproduce even better species in future generations. So, a clear understanding and modeling of foraging behavior in any of the evolutionary species leads to its application in any nonlinear system optimization algorithm. The foraging strategy of Escherichia Coli bacteria present in human intestine was introduced by Passino [12] and explained by four processes: chemotaxis, swarming, reproduction, and elimination dispersal. The characteristics of movement of bacteria in search of food can be defined in two ways, i.e., swimming and tumbling, together known as chemotaxis. A bacterium is said to be “swimming” if it moves in a predefined direction, and “tumbling” if moving in altogether different direction. In the swarming phase, and for the bacteria to reach the richest food location (i.e., for the algorithm to converge at the solution point), it is desired that the optimum bacterium till a point of time in the search period should try to attract other bacteria so that together they converge at the desired location (solution point) more rapidly. To achieve this, a penalty function based upon the relative distances of each bacterium from the fittest bacterium till that search duration, is added to the original cost function. Finally, when all the bacteria have merged into the solution point, this penalty function becomes zero. The effect of swarming is to make the bacteria congregate into groups and move as concentric patterns with high bacterial density. In regard to the reproduction phase, the original set of bacteria, after getting evolved through several chemotactic stages, reach the reproduction stage. Here, best set of bacteria (chosen out of all the chemotactic stages) get divided into two groups. The healthier half replaces with the other half of bacteria, which gets eliminated, owing to their poorer foraging abilities. This makes the population of bacteria constant in the evolution process. In the evolution process, a sudden unforeseen event can occur, which may drastically alter the smooth process of evolution and cause the elimination of the set of bacteria and/or disperse them to a new environment. Most ironically, instead of disturbing the usual chemotactic growth of the set of bacteria, this unknown event may place a newer set of bacteria nearer to the food location. From a broad perspective, elimination and dispersal are parts of the population-level long-distance motile behavior. In its application to optimization, it helps in reducing the behavior of stagnation (i.e., being trapped in a premature solution point or local optima) often seen in such parallel search algorithms. Table 1 represents the main parameters of BFO algorithm.

**Table 1:** BFO algorithm parameters [12]

Symbol	Description
*p*	Search space dimension
*S*	Total number of bacteria in the population
$N_{\text{s}}$	Maximum number of swim
$N_{\text{c}}$	Total number of chemotaxis
$N_{\text{re}}$	Maximum number of reproduction
$N_{\text{ed}}$	Maximum number of elimination and dispersal events
$P_{\text{ed}}$	Probability of the elimination and dispersal of bacterium
*C*	Step size of the bacterium tumble
*J*	Cost function value

### BFO implementation

BFO has been applied in numerous research areas. Supriyono and Tokhi [13] proposed an adaptable chemotactic step size BFO in modeling a single-link flexible manipulator system. Based on an experimental single-link flexible manipulator rig, the input–output data have been collected and employed in establishing three single-input single-output models to characterize the system. As for Kalaam et al. [14], their study considered the implementation of bacterial foraging algorithm for optimizing multiple PI controllers’ design variables in a cascaded structure. Four proportional-integral (PI) controllers were employed for controlling a grid-connected photovoltaic (PV) system. Simulation results revealed that the optimized design values improved the system performance. The performance of utilizing bacterial foraging algorithm (BFO) on an intelligent fuzzy logic controller for a unicycle class of differential drive robot on an irregular rough terrain was investigated by Almeshal et al. [15]. Based on simulation results, the BFO algorithm improved the control method and a satisfactory convergence has been achieved. Nasir et al. [16] focused on improving the performance of BFO by proposing novel adaptive bacteria foraging algorithms based on index of iteration, index of chemotaxis, and fitness value to overcome the oscillations in the convergence graph caused by the constant step size and to speed up the convergence in the case of using small step size. The developed algorithms have been examined with multiple unimodal and multimodal standard benchmark functions. Considering different dimensions and fitness landscapes, simulation results revealed the outperformance of the developed algorithms based on convergence speed and fitness accuracy. On the other hand, Agouri et al. [17] proposed a control strategy for a two-wheeled robot with an extendable IB using quadratic adaptive bacterial foraging algorithm (QABFA). Nasir et al. [18] focused on improving the spiral dynamic algorithm by considering both elimination and dispersal phases of bacterial foraging algorithm. The improved SDA’s performance has been tested and implemented in fuzzy logic dynamic modeling of a twin rotor system. According to simulation results, the improved SDA converged to a far better solution compared to other implemented optimization algorithms. Considerable amount of research studies merged the concept of bacterial foraging algorithm with other algorithms and developed hybrid optimization algorithms [19, 21].

### Overview and contribution

This paper presents a bacterial foraging technique for determining the optimal parameters of a PID controller to control the stability of a five degrees-of-freedom (DOF) two-wheeled machine (TWM) developed by Goher [22].

The novel 5-DOF TWM provides payload handling in two mutually perpendicular directions while attached to the IB. Compared to existing TWRMs, this design increases both workspace and flexibility of two-wheeled machines and allows them to be employed in service and industrial robotic applications including objects assembly and material handling. Bacterial foraging algorithm’s potential, as illustrated in the literature, was a source of encouragement to examine the proposed optimization technique on the novel 5 DOF two-wheeled machine’s controller in order to improve the system’s stability performance.

### Paper organization

The rest of the paper is organized as follows: “Bacterial foraging optimization (BFO) algorithm” section presents an overview of the BFO algorithm and a rationale about the implementation on various nonlinear dynamic systems, the system description machine is presented in “TWRM system description,” and “System modeling” sections present the previously developed mathematical model using Lagrangian approach. “Control system design” section describes the control system design and the implementation of bacterial foraging optimization technique including various courses of motion and testing of the robustness of the control approach. At last, the main conclusions of the paper are presented in “Conclusions” section.

## Methods

### TWRM system description

**Fig. 1:**
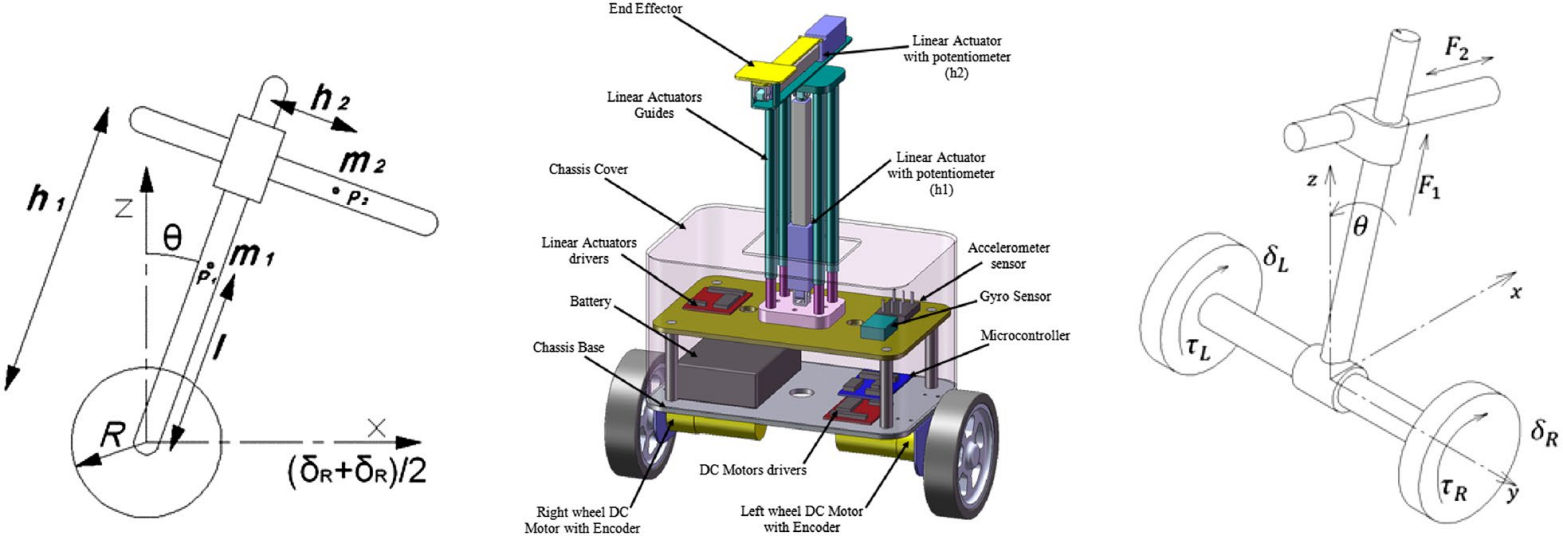
System schematic diagrams

**Fig. 2:**
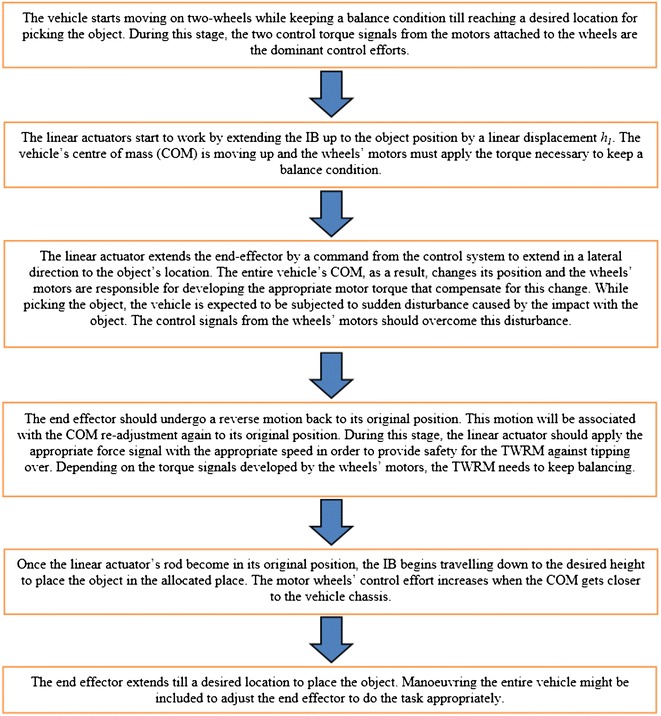
TWRM courses of motion

 Figure 1 illustrates the schematics diagram of the two-wheeled robotic machine (TWRM). The proposed system consists of a chassis with center of gravity at point *P*_1_ and the mass of the linear actuators with center of gravity at point *P*_2_. The coordinates of *P*_1_ and *P*_2_ will change as long as the robot maneuvers away from its initial position along the *X* axis. The two motors attached to each wheel are in charge of providing the necessary torque, $\tau_{\text{R}}$ and $\tau_{\text{L}}$, for controlling the TWRM. For enabling the control strategy to maintain the two-wheeled machine’s position at the upright position continuously, the system is equipped with both accelerometer and gyroscope sensors that provide multiple state variables information at any given time. The system design provides compactness with offering proper rooms for system electronics and accessories. Other targeted features include a lightweight structure without affecting the robot stiffness and a symmetrical mass distribution for the entire robot parts and components at initial position. With respect to the *X* and *Z* axes, four types of translations define the system’s DOFs: the attached payload linear displacement in vertical and horizontal directions $h_{1}$ and $h_{2}$, respectively, and the angular displacement of the angular rotation of the right and left wheels $\delta_{\text{R}}$ and $\delta_{\text{L}}$, respectively. The tilt angle $\theta$ of the intermediate body around the vertical *Z* axis is considered as the fifth remaining DOF. Consider a picking up and placing scenario, as an application of the proposed configuration, with the vehicle’s course of motion illustrated in Fig. 2.

The activation of each of the TWRM actuators against each subtask and the DOFs involved in each process, for the previously mentioned picking and placing scenario, is illustrated in Table 2. Due to the continuous variation in the location of the center of mass (COM) as well as the external disturbances taking place during the picking and/or placing the object task, the system wheels’ motors remain activated during the whole process. Developing a sufficient torque signal from both wheels’ motors is mandatory for maintaining the balancing of the TWRM in the upright vertical position. As for the linear actuators, activating them counts on the selected subtask (i.e., picking, placing, both tasks). As a main part of the control algorithms, switching mechanisms are designed to define the period of engagement of each individual actuator in service.

**Table 2:** Subtasks against engagement of individual actuators

Subtask	Associated DOFs	Right motor $\tau_{\text{R}}$	Left motor $\tau_{\text{L}}$	Linear actuator I, $F_{1}$	Linear actuator II, $F_{2}$
Moving and picking	$\delta_{\text{L}}$, $\delta_{\text{R}} , \theta$	✓	✓	✕	✕
IB extension	$\delta_{\text{L}}$, $\delta_{\text{R}} , \theta , h_{1}$	✓	✓	✓	✕
Extension: end effector	$\delta_{\text{L}}$, $\delta_{\text{R}} , \theta , h_{2}$	✓	✓	✕	✓
Reverse: end effector	$\delta_{\text{L}}$, $\delta_{\text{R}} , \theta , h_{2}$	✓	✓	✕	✓
IB contraction	$\delta_{\text{L}}$, $\delta_{\text{R}} , \theta , h_{1}$	✓	✓	✓	✕
Placing the object	$\delta_{\text{L}}$, $\delta_{\text{R}} , \theta , h_{2}$	✓	✓	✕	✕

## System modeling

Among the diverse methods of deriving the equations of motion, and due to the fact that it is a powerful approach, Lagrangian modeling approach is employed to model the TWRM. Based on the system schematic diagrams illustrated earlier, the vehicle’s mathematical model is derived by relating the system’s kinematics to the torques/forces applied (details of the model derivation can be found in the work of Goher [22]). The system’s mathematical model is presented as five nonlinear-coupled differential equations as follows:

For the vertical linear link displacement ($h_{1}$):

1 $$\frac{1}{2}m_{2} \left( {2g\cos \theta - 2h_{1} \dot{\theta }^{2} - 4\dot{h}_{2} \dot{\theta } - 2h_{2} \ddot{\theta } + 2\ddot{h}_{1} + (\ddot{\delta }_{\text{R}} + \ddot{\delta }_{\text{L}} )\sin \theta } \right) = F_{1} - \mu_{1} \dot{h}_{1}$$

For the horizontal link displacement ($h_{2}$):

2 $$\frac{1}{2}m_{2} \left( {2g\sin \theta + 2h_{2} \dot{\theta }^{2} - 4\dot{h}_{1} \dot{\theta } - 2h_{1} \ddot{\theta } - 2\ddot{h}_{2} - (\ddot{\delta }_{\text{R}} + \ddot{\delta }_{\text{L}} )\cos \theta } \right) = F_{2} - \mu_{2} \dot{h}_{2}$$

For the angular displacement of the left wheel ($\delta_{\text{L}}$):

3 $$\frac{1}{2}m_{1} \left( {\frac{1}{2}\ddot{\delta }_{\text{R}} + \frac{1}{2}\ddot{\delta }_{\text{L}} - l\dot{\theta }^{2} \sin \theta + l\ddot{\theta }\cos \theta } \right) + \frac{1}{2}m_{2} \left( {\ddot{h}_{1} \sin \theta + 2\dot{h}_{1} \dot{\theta }\cos \theta - h_{1} \dot{\theta }^{2} \sin \theta + h_{1} \ddot{\theta }\cos \theta + \ddot{h}_{2} \cos \theta - 2\dot{h}_{2} \dot{\theta }\sin \theta - h_{2} \dot{\theta }^{2} \cos \theta - h_{2} \ddot{\theta }\sin \theta + \frac{1}{2}\ddot{\delta }_{\text{R}} + \frac{1}{2}\ddot{\delta }_{\text{L}} } \right) + 2m_{\text{w}} \ddot{\delta }_{\text{L}} + 2J_{\text{w}} \frac{{\ddot{\delta }_{\text{L}} }}{{R^{2} }} = \tau_{\text{L}} - \mu_{\text{w}} \left( {\frac{{\dot{\delta }_{\text{L}} }}{{R^{2} }}} \right) - \mu_{\text{c}} \dot{\delta }_{\text{L}}$$

For the angular displacement of the right wheel ($\delta_{\text{R}}$):

4 $$\frac{1}{2}m_{1} \left( {\frac{1}{2}\ddot{\delta }_{\text{R}} + \frac{1}{2}\ddot{\delta }_{\text{L}} - l\dot{\theta }^{2} \sin \theta + l\ddot{\theta }\cos \theta } \right) + \frac{1}{2}m_{2} \left( {\ddot{h}_{1} \sin \theta + 2\dot{h}_{1} \dot{\theta }\cos \theta - h_{1} \dot{\theta }^{2} \sin \theta + h_{1} \ddot{\theta }\cos \theta + \ddot{h}_{2} \cos \theta - 2\dot{h}_{2} \dot{\theta }\sin \theta - h_{2} \dot{\theta }^{2} \cos \theta - h_{2} \ddot{\theta }\sin \theta + \frac{1}{2}\ddot{\delta }_{\text{R}} + \frac{1}{2}\ddot{\delta }_{\text{L}} } \right) + 2m_{\text{w}} \ddot{\delta }_{\text{R}} + 2J_{\text{w}} \frac{{\ddot{\delta }_{\text{R}} }}{{R^{2} }} = \tau_{\text{R}} - \mu_{\text{w}} \left( {\frac{{\dot{\delta }_{\text{R}} }}{{R^{2} }}} \right) - \mu_{\text{c}} \dot{\delta }_{\text{R}}$$

For the tilt angle of the intermediate body ($\theta$):

5 $$2m_{2} \dot{\theta }(\dot{h}_{2} h_{2} + \dot{h}_{1} h_{1} ) + \frac{1}{2}m_{2} (h_{1} \cos \theta - h_{2} \sin \theta )(\ddot{\delta }_{\text{R}} + \ddot{\delta }_{\text{L}} ) + \frac{1}{2}m_{1} l\cos \theta (\ddot{\delta }_{\text{R}} + \ddot{\delta }_{\text{L}} ) - m_{2} g(h_{1} \sin \theta + h_{2} \cos \theta ) + \ddot{\theta }\left( {J_{1} + J_{2} + m_{1} l^{2} + m_{2} h_{2}^{2} + m_{2} h_{1}^{2} } \right) + 2m_{2} h_{1} h_{2} - m_{1} gl\sin \theta = 0$$

## Control system design

In the previous section, the TWRM equations of motion have been developed. This section focuses on testing the derived model to obtain its response and to manage to control it by implementing and comparing between different control strategies for the purpose of obtaining a satisfactory response for the system. Examining the behavior of the developed model for the TWRM requires investigating the system’s open-loop response. The derived model is simulated in MATLAB/Simulink^®^ environment by utilizing the simulation parameters shown in Table 3. In a previous study conducted by Goher [22], it was revealed that the system response is unstable nonlinear system. Based on that, a closed-loop system is substantial for stabilizing the TWRM and improving the system’s performance. In this work, five decoupled feedback control loops have been used throughout the work. The developed control strategy, based on loops decoupling, ensures separation of the system dynamics due to the high frequency range (tilt angle) from the dynamics of low frequency range (motion of the intermediate body). The two feedback control loops occupy separate ranges of dynamics, low frequency and high frequency with tilt angle over higher frequency range and motion of intermediate body over lower frequency range, and hence, the decoupling approach is reasonable to use and apply separate control loops.

**Table 3:** System simulation parameters

Parameter	Description	Value	Unit
$m_{1}$	Mass of the chassis	1	kg
$m_{2}$	Mass of the linear actuators	0.6	kg
$m_{\text{w}}$	Mass of wheel	0.14	kg
$g$	Gravitational acceleration	9.81	m/s^2^
$l$	Distance of chassis’s center of mass for wheel axle	0.14	m
$R$	Wheel radius	0.05	m
$J_{1}$	Rotation inertia of chassis	0.068	kg m^2^
$J_{2}$	Rotation inertia of moving mass	0.0093	kg m^2^
$J_{\text{w}}$	Rotation inertia of a wheel	0.000175	kg m^2^
$\mu_{\text{c}}$	Coefficient of friction between chassis and wheel	0.1	N s/m
$\mu_{\text{w}}$	Coefficient of friction between wheel and ground	0	N s/m
$\mu_{1}$	Coefficient of friction of vertical linear actuator	0.3	N s/m
$\mu_{2}$	Coefficient of friction of horizontal linear actuator	0.3	N s/m

The friction at the mating surfaces has been simplified for the chassis–wheel, wheel–ground interaction and in the linear actuator to follow Coulomb frictional model. The values of the coefficients have been selected depending on the type of surfaces. The selected constant values are assumed to be valid under all working conditions of the vehicle and the actuators. This did not take into account variations in speed, path configuration, terrain profile, etc. The constant values have been used to validate the system model. However, modeling interactions between surfaces need to be investigated for various surfaces, various terrain profiles, and various operation conditions of the vehicle.

### BFO-optimized PID control design

In this part, bacterial foraging optimization technique is applied on the system in order to optimize the PID controller gains employed in a previous research study [22] by maintaining the system in the upright position and to counteract the disturbances occurring in different motion scenarios.

#### Optimization algorithm objective functions and constraints

**Fig. 3:**
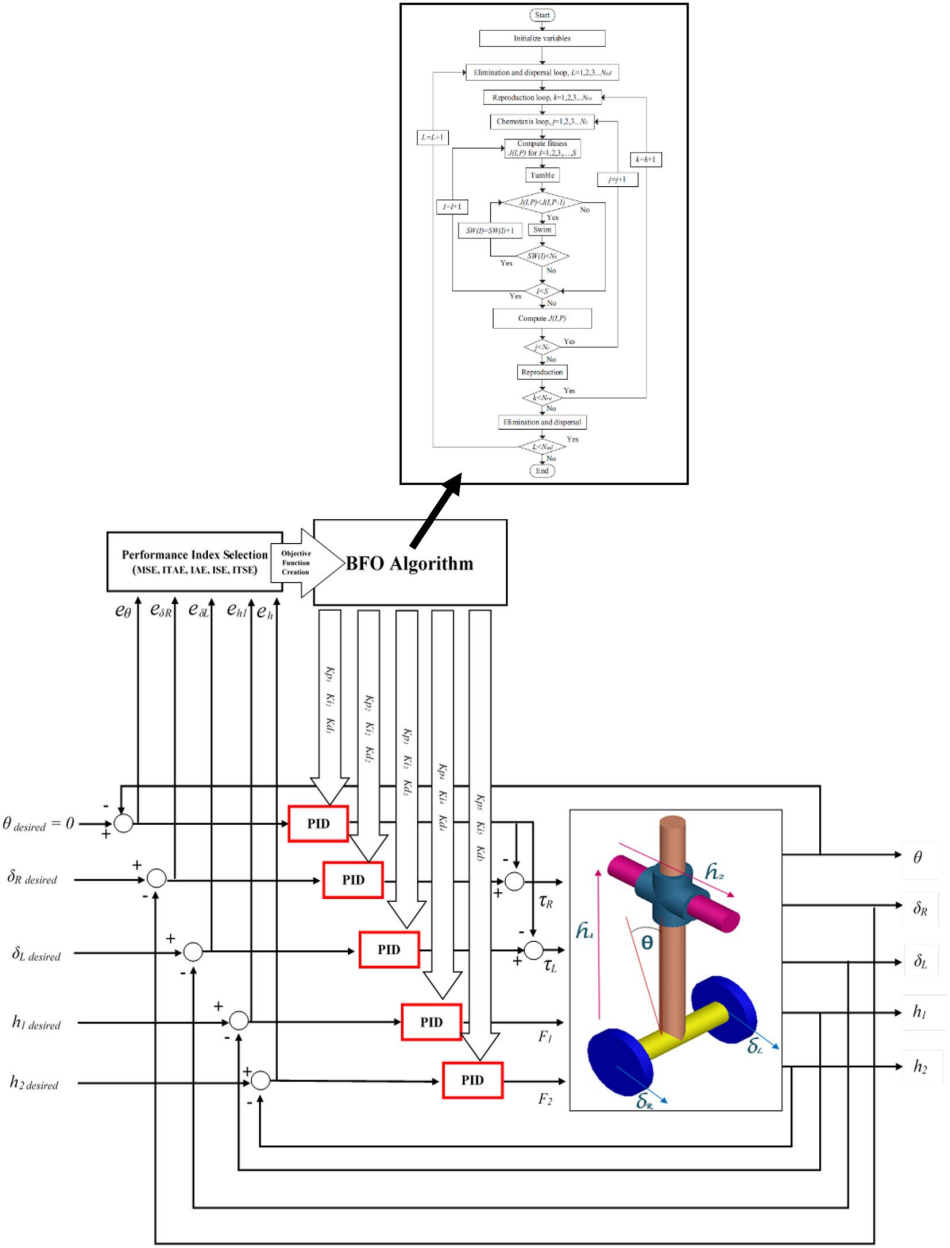
TWRM’s BFO-optimized PID control scheme

**Fig. 4:**
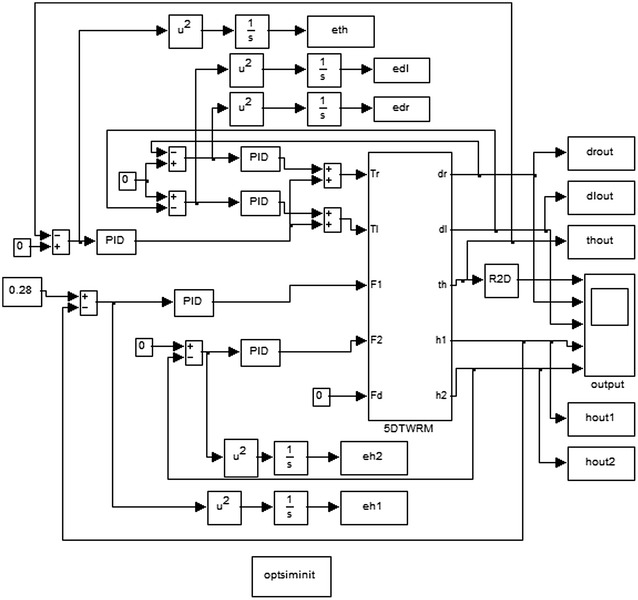
Simulink^®^ model of the BFO-optimized PID controller

The most critical step in applying optimization techniques is to choose the objective functions that are used to evaluate fitness function. The objective functions can be created using performance indices functions to evaluate the errors of the controlled loops. These performance indices used to optimize the errors of the system are: mean of the squared error (MSE), integral of time multiplied by absolute error (ITAE), integral of absolute magnitude of the error (IAE), integral of the squared error (ISE), and time multiplied by the squared error (ITSE) to minimize the error signals and compare them to find the most suitable one [23]. The TWRM’s bacterial foraging-optimized PID control scheme schematic description is demonstrated in Fig. 3. Figure 4 shows the MATLAB/Simulink model of the PID controller optimized by BFO built to determine the errors using Eqs. (6)–(10). The optimized PID controller is used to minimize the error signals by minimizing the value of the objective functions of performance indices defined as follows:

6 $${\text{MSE}} = \frac{1}{t}\int\limits_{0}^{\tau } {\left( {e(t)} \right)^{2} } {\text{d}}t,$$

7 $${\text{ITAE}} = \int\limits_{0}^{\tau } {t\left| {e(t)} \right|} {\text{d}}t,$$

8 $${\text{IAE}} = \int\limits_{0}^{\tau } {\left| {e(t)} \right|} {\text{d}}t,$$

9 $${\text{ISE}} = \int\limits_{0}^{\tau } {e(t)^{2} } {\text{d}}t,$$

10 $${\text{ITSE}} = \int\limits_{0}^{\tau } {te(t)^{2} } {\text{d}}t,$$

where *e*(*t*) is the error signal in time domain.

## Results and Discussions

### Implementation of BFO-PID algorithm

The behavior of the robotic machine was observed for the tilt angle of the entire vehicle, angular displacements of the two wheels, and linear displacements of the linear actuators using different motion scenarios.

#### Payload free motion (*h*_1_ = *h*_2_ = 0)

**Fig. 5:**
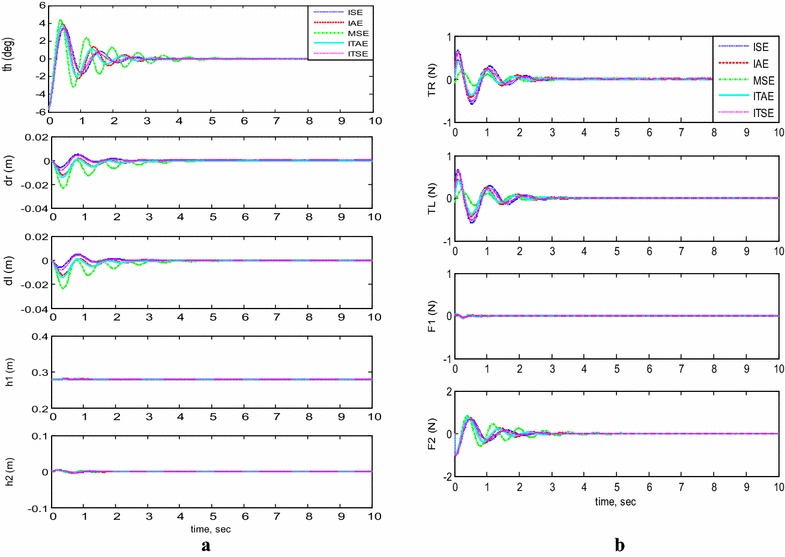
System outputs and inputs (*h*_1_ = *h*_2_ = 0). **a** System outputs, **b** system inputs

Figure 5a, b demonstrates the simulation results of the system performance and inputs control signals. The system is considered to start initially at $\theta = - 5^\circ$ and neglecting the effect of both linear actuators, $h_{1}$ and $h_{2}$, by setting them to zero during the system stabilization. Table 4 summarizes the performance of the system by determining the overshoot, settling time, rise time, and peak time values of each methods of error determinations. PID controller optimized by MSE gives a value of 46.6% of overshoot which is the biggest value for the overshoot, followed by 34.8% overshoot value for ITSE. The controller optimized by IAE gives the best value with minimum overshoot, 27.9%, where ISE and ITAE give good overshoot percentages for 33.8 and 29.3%, respectively. In summary, it is clear that BFO reduces the percent overshoot, especially if optimized by IAE criterion. As for settling time, it can be seen that the settling time values of BFO vary in the range of 0.78 s for IAE to 1.87 s for BFO optimized by MSE. The best method to optimize the settling time is by using the IAE. From Table 4, the best rise time is given by MSE (0.19 s) and the worst one is ITAE (0.248 s). All other BFO methods produce almost the same value. But because BFO results are not so different, it cannot be concluded that BFO can optimize the rise time. As in rise time values, the peak time values show almost the same values for all methods with small variation between them. All other BFO error methods produce peak time between 0.35 and 0.46 s. The best values are given by BFO optimized by MSE and ISE, 0.35 s. In short, the best optimized PID controller employed is the one optimized by IAE for the low percent overshoot and minimum settling time.

**Table 4:** System performance using different performance indices

Performance index	Percent overshoot	Settling time (s)	Rise time (s)	Peak time (s)
ISE	33.8	0.9650	0.2150	0.3950
IAE	27.9	0.7800	0.2300	0.4400
MSE	46.6	1.8720	0.1970	0.3540
ITAE	29.3	0.8080	0.2480	0.4610
ITSE	34.8	1.0140	0.2230	0.4110

#### Payload vertical movement only

**Fig. 6:**
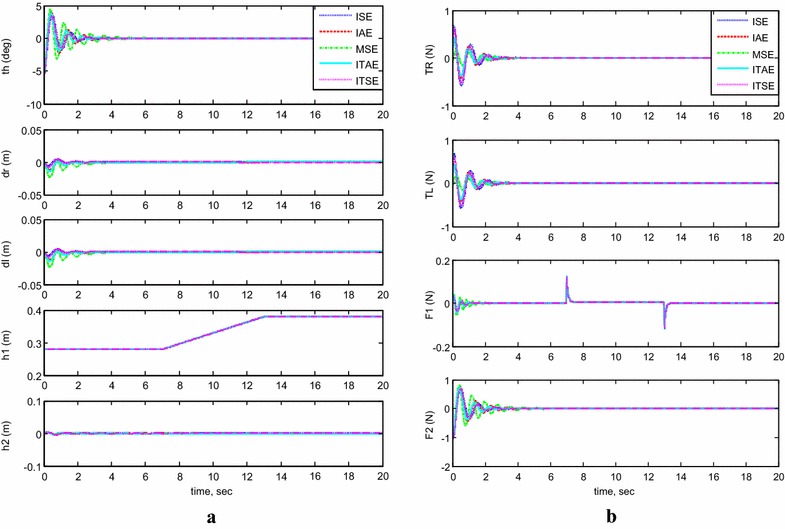
System outputs and inputs, *payload vertical movement only.* **a** System outputs, **b** system inputs

In addition, the system stability was examined for each BFO optimization criterion against the vertical linear motion of its center of mass. Considering the following initial conditions: $\theta = - \;5^\circ$, $h_{1} = 0.28\,{\text{m}}$ and neglecting the effect of the horizontal linear actuators $h_{2}$, Fig. 6 demonstrates outputs and inputs simulation of the system in the case where the payload is kept fixed for a period of 12 s from the start of the simulation and then activated to move in a vertical direction along the IB for a distance of 10 cm before settling again at a height of around 38 cm from the chassis of the vehicle. It is clear, since no disturbance occurred in the stabilization condition of the IB, that the control scheme was robust and maintained the system’s stability against the motion of $h_{1}$. Out of the five methods, the BFO optimized by IAE performed better than the other methods.

#### Payload horizontal movement only

**Fig. 7:**
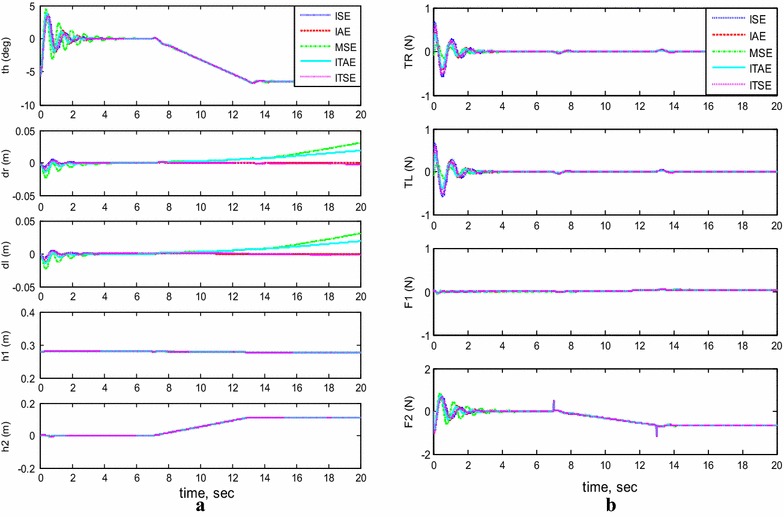
System outputs and inputs, *payload horizontal movement only.* **a** System outputs, **b** system inputs

For completing the picking/placing handling task, the TWRM is allowed to transfer the picked payload in a horizontal direction parallel to the chassis’ axis. Figure 7 illustrates the output response of the system with moving the horizontal linear actuator only and its effects on the performance of the system stability. The carried load is kept stationary at a height of 28 cm. As for the horizontal actuator, it is permitted to orient horizontally for a distance of 7 cm before settling again at a fixed position. The model’s initial conditions are set as follows: $\theta = 5^\circ$, $h_{1} = 0.28\,{\text{m}}$, and $h_{2} = 0\,{\text{m}}$. As can be seen from this scenario’s simulation results, for the MSE and ITAE criterion, the system stability was affected by the activation of the horizontal actuator and the TWRM keeps moving instead of maintaining its position. On the other hand, the remaining methods produce better performance and good robust against the movement of the horizontal actuator.

#### Simultaneous vertical and horizontal motion (*h*_1_ and *h*_2_ ≠ 0)

**Fig. 8:**
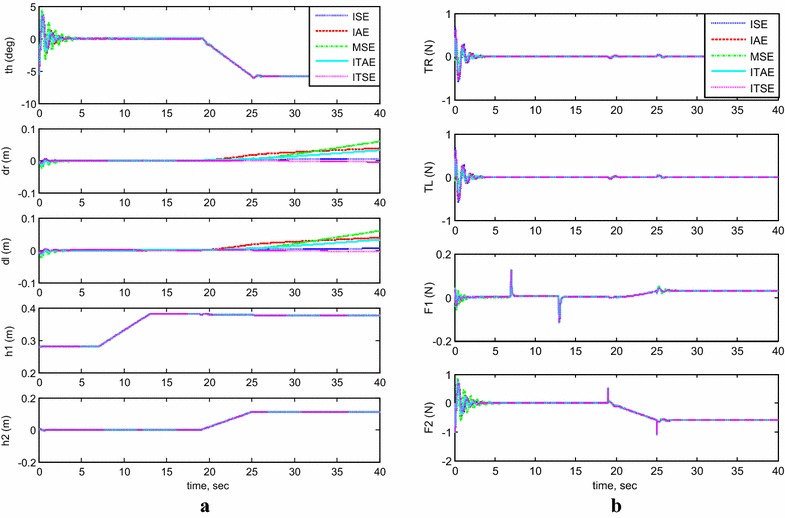
System outputs and inputs (*h*_1_ and *h*_2_ ≠ 0). **a** System outputs, **b** system inputs

Figure 8a, b demonstrates the system’s output response with moving the two linear actuators simultaneously and their effects on the system’s stability performance. The initial conditions are set to $\theta = - \;5^\circ$, $h_{1} = 0.28\,{\text{m}}$, and $h_{2} = 0\,{\text{m}}$. The system, without any interruptions, remains stable during the operation of initiating the vertical linear actuator. However, once the horizontal actuator starts to extend its rod, for both MSE and ITAE criterions, the system stability was affected and the TWRM wheels keep maneuvering instead of preserving its position. This issue did not appear in the remaining methods’ simulation results and produced good robust and better performance. Another phenomenon has been noticed, while the horizontal actuator activates. The IB tilts to the opposite direction of the horizontal actuator’s extension with a steady inclination angle of around $7^\circ$ for withstanding the change in the COM’s position.

#### Trajectory of a 1-m straight-line motion

**Fig. 9:**
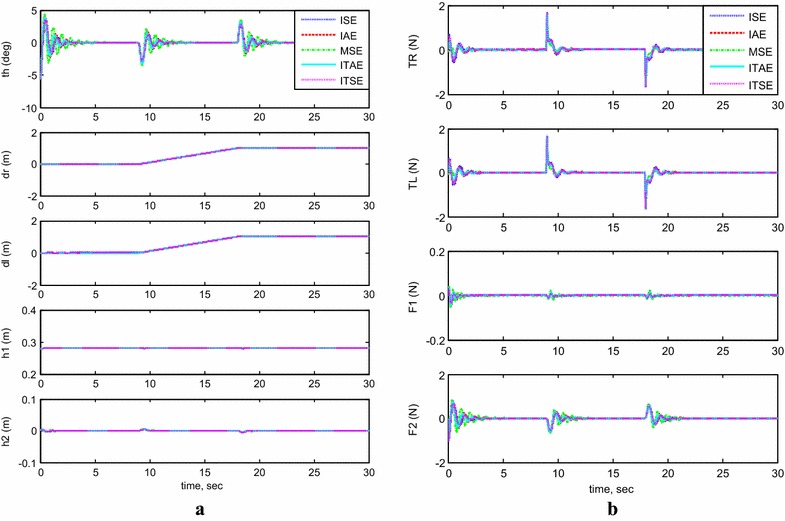
System outputs and inputs, 1-m straight-line motion. **a** System outputs, **b** system inputs

The system stability was examined during moving the TWRM in a straight line for 1 m after balancing the robot in the upside position, and the simulation results are shown in Fig. 9. Referring to Fig. 9a, the control scheme, including the five tested criterions, was capable to counter the occurred disturbances caused by the wheels’ motors’ activation at the beginning (8 s) and the end (18 s) of the straight-line motion. As can be observed in Fig. 9b, the maximum control effort spent for maneuvering the system in a 1-m straight-line trajectory, compared to the other criterions, was noted for the ITSE method, around 1.8 N.

#### Control system robustness

**Fig. 10:**
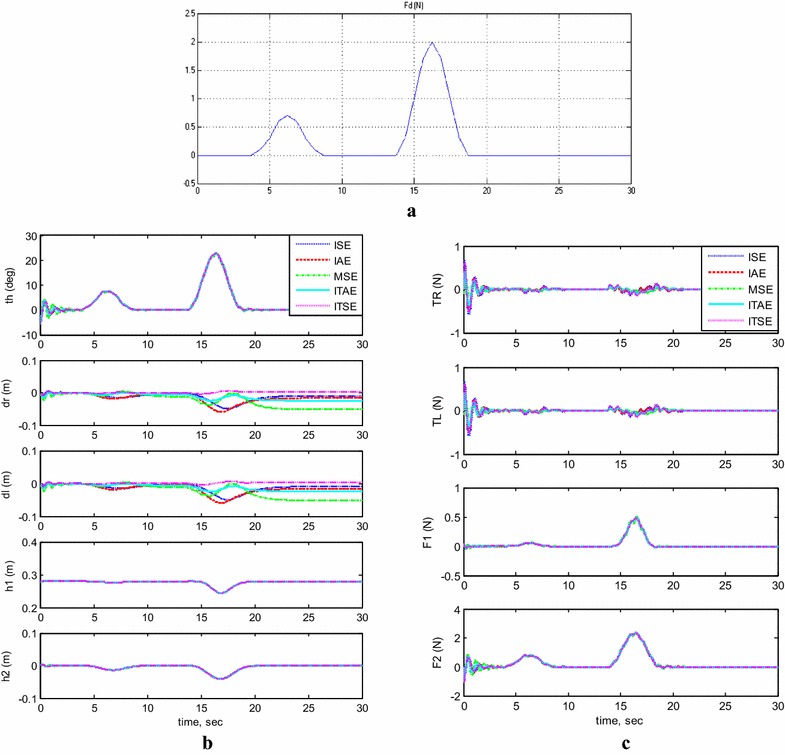
System outputs and inputs, with disturbance force. **a** The applied disturbance, **b** system outputs, **c** system inputs

Moreover, the system stability was examined against the effect of disturbance forces illustrated in Fig. 10a and the simulation results are shown in Fig. 10b, c. As it is shown that disturbance force affects the system, but the controller reacts against this force to stabilize the system. However, the controller resulted from MSE shows that the displacement of the system is effected with slight change, while the other optimized controllers show better performance.

### Comparison between implementation of PID and BFO-optimized PID

The boundary limits of the controller gain parameters for each loop that were applied in MATLAB/Simulink environment were obtained by trial and error. In this section, the authors carry out a comparison of the system response based on the implementation of two approaches. The control gain parameters employed in each control loop for the control schemes in order to achieve a satisfactory system performance are listed in Table 5.

**Table 5:** Gain values for different control schemes

Loop	Output parameter	Gain parameters	PID + switching	BFO + switching		
				Lower boundary	Calculated gain	Upper boundary
Loop 1	$\theta$	*Kp*_1_	80	−50	−1.733	50
		*Kd*_1_	9	−10	−0.0693	10
		*Ki*_1_	0.02	−0.1	0.0835	0.1
Loop 2	$\delta_{\text{R}}$	*Kp*_2_	80	−20	10.255	20
		*Kd*_2_	75	−20	0.016	20
		*Ki*_2_	0.05	−20	15.05	20
Loop 3	$\delta_{\text{L}}$	*Kp*_3_	80	−20	10.255	20
		*Kd*_3_	75	−20	0.016	20
		*Ki*_3_	0.05	−20	15.05	20
Loop 4	$h_{1}$	*Kp*_4_	8	−20	10.3279	20
		*Kd*_4_	10	−10	7.3378	10
		*Ki*_4_	0.01	−0.1	0.013	0.1
Loop 5	$h_{2}$	*Kp*_5_	27	−60	50.1502	60
		*Kd*_5_	32	−50	30.7237	50
		*Ki*_5_	0.05	−0.1	0.027	0.1

**Fig. 11:**
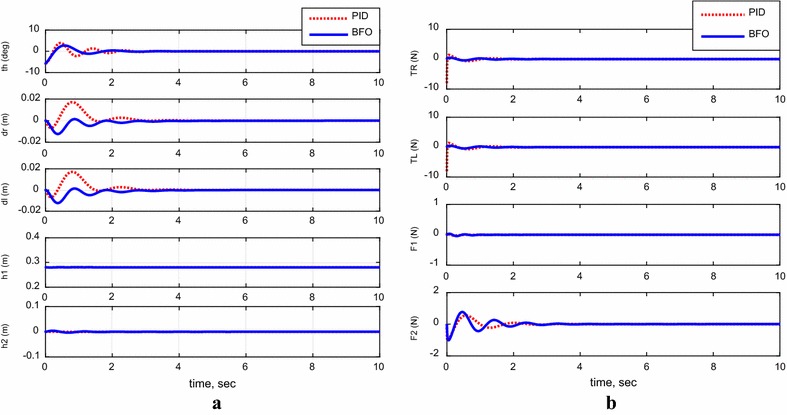
System outputs and inputs, payload free movement (*h*_1_ = *h*_2_ = 0). **a** System outputs, **b** system inputs

**Fig. 12:**
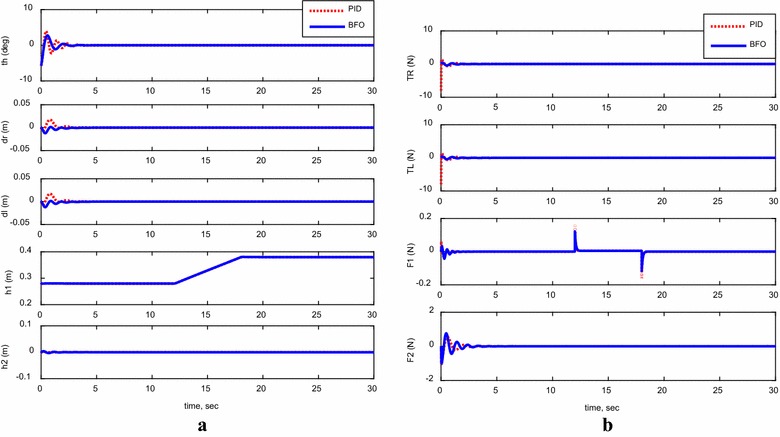
System outputs and inputs, *payload vertical movement only.* **a** System outputs, **b** system inputs

**Fig. 13:**
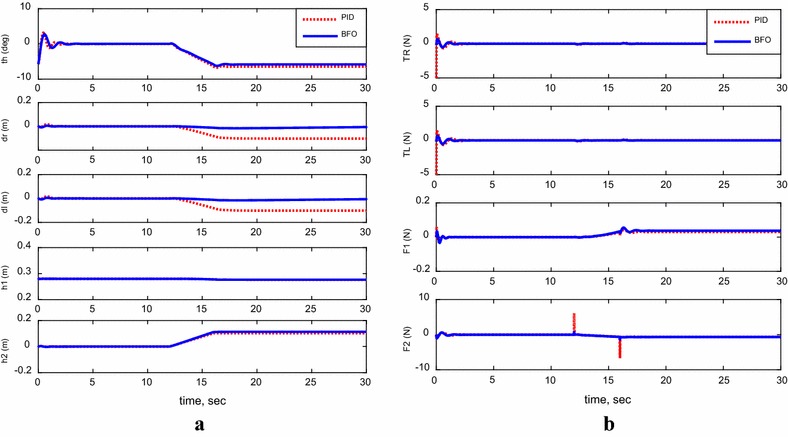
System outputs and inputs, *payload horizontal movement only*. **a** System outputs, **b** system inputs

**Fig. 14:**
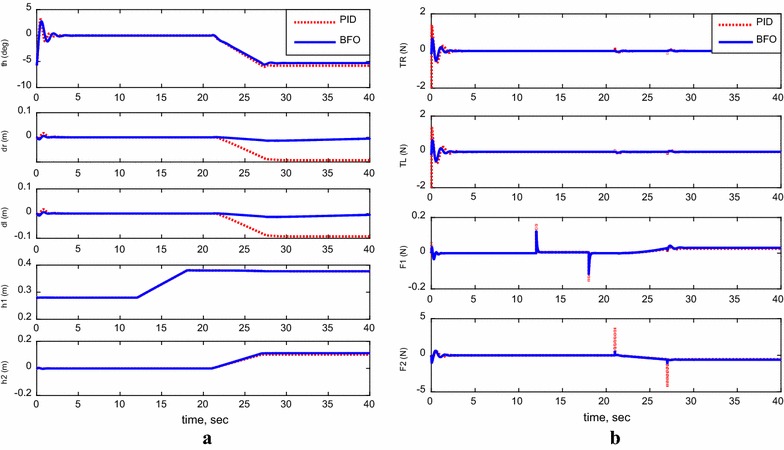
System outputs and inputs, simultaneous vertical and horizontal motion (*h*_1_ and *h*_2_ ≠ 0). **a** System outputs, **b** system inputs

**Fig. 15:**
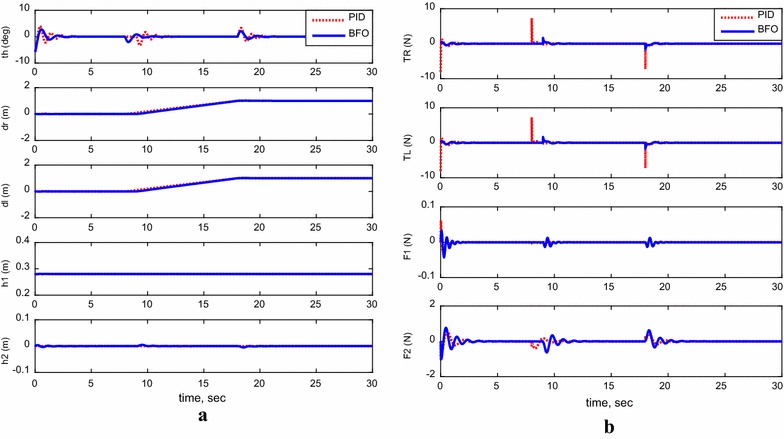
System outputs and inputs, a 1-m straight-line motion. **a** System outputs, **b** system inputs

For the following cases, payload free movement, payload vertical movement only, payload horizontal movement only, simultaneous horizontal and vertical motion, and 1-m straight-line vehicle motion, Figs. 11, 12, 13, 14, and 15 demonstrate the output results of the simulated TWRM model and the applied control effort. It is clear, from the previous figures, that the optimized controller by BFO provides better performance for the system and minimizes the applied force demanded for the TWRM stabilization process. Observing the payload free movement (*h*_1_ = *h*_2_ = 0) scenario, as an example of how the BFO-optimized PID controller performance is better way than the PID, Table 6 illustrates a system performance comparison between the previously mentioned controllers in terms of overshoots, settling time, peak time, and rise time.

**Table 6:** Comparison between the system’s performance using PID and BFO

Control	Percent overshoot	Settling time (s)	Peak time (s)	Rise time (s)
PID	48.1	2.2870	0.5710	0.2790
BFO	27.9	0.7800	0.4400	0.2300

Starting with system overshoot, the BFO-optimized PID control scheme provides better overshoot value (27.9%), which is much less than the PID-recorded overshoot value by almost 42%. Moving to settling time, it is observable that by implementing the PID control strategy the system takes around 2.3 s to settle, which is greater than the BFO-optimized PID control method’s settling time (0.78 s). Therefore, the BFO-optimized PID control strategy optimizes the settling time. As in peak and rise time values, a slight reduction has been noticed when the BFO-optimized PID control method is implemented on the TWRM model and it can be concluded that the BFO-based method produces both peak and rise time values better than the PID controller.

From the scenarios where the horizontal linear actuator activates, payload horizontal movement only case (Fig. 13) and simultaneous horizontal and vertical motion case (Fig. 14), the issue of the TWRM’s continuous movement that results from the activation of the horizontal actuator has been compensated by the implementation of bacterial foraging algorithm. For the response associated with the PID control scheme, the activation of the horizontal actuator affects the system’s stability and allows the TWRM to move 10 cm away from its original location ($\delta_{\text{R}}$ = −0.1 m, $\delta_{ \, }$ = −0.1 m). However, the BFO-optimized PID controller produces a satisfactory performance and robustness against the horizontal linear actuator’s movement. In general, the BFO-optimized PID control method produces much better system performance and optimized behavior than PID control scheme, where the optimum controller values are resultant from the IAE criterion.

#### Investigating control system robustness

**Fig. 16:**
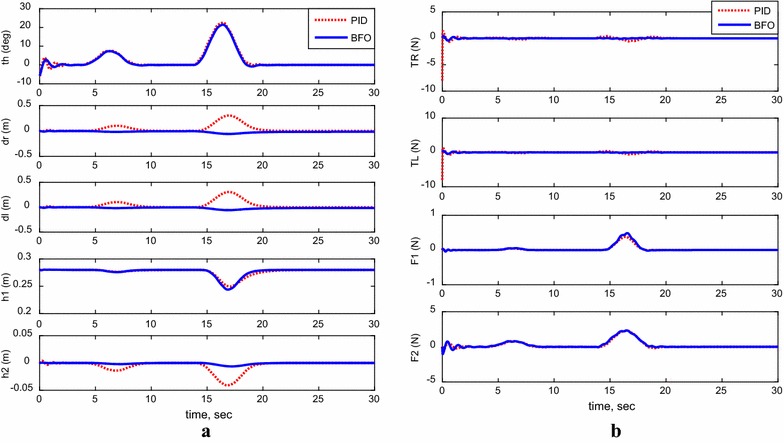
System outputs and inputs with a disturbance. **a** System outputs, **b** system inputs

Considering the same disturbance force applied earlier on the system, shown in Fig. 10a, the system’s robustness was examined for the two control methods and the performance of the system is demonstrated in Fig. 16. As can be observed, for both control methods and in few seconds, the system bounced back to its stability region around the vertical axis. However, the PID performance was not sufficient to withstand the effect of disturbance on the TWRM wheels’ displacement ($\delta_{R}$, $\delta_{L}$) and the horizontal linear actuator displacement (*h*_2_). As a matter of fact, the bacterial foraging-optimized PID control method surpassed the PID control scheme in terms of performance, robustness, and instability minimization.

The upper and lower boundaries, shown in Table 5, are associated with the BFO-optimized control scheme with switching mechanism. Those parameters are required by the BFO algorithm in order to calculate the optimal gain values.

## Conclusions

A bacterial foraging optimization algorithm for determining the optimal parameters of PID controller employed for controlling the stability of a novel five DOF two-wheeled robotic machine has been presented in this paper. Lagrangian approach has been utilized for deriving the TWRM’s nonlinear mathematical model that has been simulated in MATLAB/Simulink^®^ environment. Furthermore, the stability of the two-wheeled machine was examined against different motion scenarios which include payload free movement, payload vertical movement only, payload horizontal movement only, simultaneous horizontal and vertical motion, and 1-m straight-line vehicle motion. In addition, the system’s stability was examined against disturbance forces to examine the controller robustness. It is clear that the bacterial foraging optimization applied in PID controller improves the system performance compared to the PID method. This was visualized by the reduction in the rise time, settling time, and percent overshoot. Further studies will consider implementing and comparing between various optimization techniques [i.e., particle swarm optimization algorithm, spiral dynamics algorithm, genetic algorithm] for optimizing the 5 DOF TWM’s PID controller gains in order to obtain the optimum control scheme that provides the best system stabilization performance.
